# Mass spectrometry-based human spatial omics: fundamentals, innovations, and applications

**DOI:** 10.1186/s12929-026-01219-0

**Published:** 2026-02-06

**Authors:** Ching-Chia Yang, Ching-Ya Lin, Hsin-Yo Yuan, Hsuan-Cheng Huang, Hsueh-Fen Juan

**Affiliations:** 1 Department of Life Science, National Taiwan University, Taipei, 106 Taiwan; 2 Center for Computational and Systems Biology, National Taiwan University, Taipei, 106 Taiwan; 3 Graduate Institute of Biomedical Electronics and Bioinformatics, National Taiwan University, Taipei, 106 Taiwan; 4 Institute of Biomedical Informatics, National Yang Ming Chiao Tung University, Taipei, 112 Taiwan; 5 Center for Advanced Computing and Imaging in Biomedicine, National Taiwan University, Taipei, 106 Taiwan; 6 Institute of Atomic and Molecular Sciences, Academia Sinica, Taipei, 106 Taiwan

**Keywords:** Mass spectrometry, Spatial omics, Multimodal data integration, Biomarker discovery, Precision medicine

## Abstract

Mass spectrometry-based spatial omics is a powerful approach for visualizing the spatial organization of proteins, metabolites, lipids, and other biomolecules in situ, combining the molecular depth of mass spectrometry with spatially resolved imaging. This systematic review traces the rapid technological and computational evolution of this field, including innovations in mass spectrometry imaging (MSI), labeling-based approaches, and proximity labeling techniques. It also highlights recent advances that enhance spatial resolution, expand molecular coverage, and enable deep molecular characterization and review analytical pipelines that integrate deep learning, cross-modality registration, and cloud-optimized data formats. From the multimodal and practical perspective, the integration of MSI with other spatial omics platforms and its transformative applications in tumor microenvironment profiling, neurodegenerative disease, developmental biology, biomarker discovery, and precision medicine are discussed. Finally, this review outlines challenges and opportunities, emphasizing the need for standardization, clinical validation, and interpretable artificial intelligence to enable broader adoption. These advances position MS-based spatial omics as a foundational pillar for multimodal spatial biology and personalized healthcare.

## Introduction

All biological processes occur within well-defined spatial arrangements. In living organisms, where the cells are, how they interact with each other, and what proteins they produce are fundamental in determining their functions and fates. At the cellular level, the localization of proteins, metabolites, and lipids dictates where biochemical reactions occur and which pathways are engaged to maintain homeostasis. At the tissue level, the neighborhood of distinct cell types and their molecular crosstalk coordinate development and immunity, while disorganization or molecular mislocalization can lead to diseases ranging from cancer to neurodegenerative disorders. Unraveling these spatial relationships, both between cells and inside single cells, is therefore essential for understanding physiology and disease.

Efforts to chart molecular maps began with immunofluorescence in the 1940s, enabling visualization of single proteins inside cells and laying the groundwork for further immunohistochemistry (IHC) technology, which is fundamental for constructing atlases of human proteomes [1–5]. Later, in situ hybridization (ISH) broadened the range to the spatial characterization of specific nucleic acid targets, evolving to next-generation sequencing (NGS)-based spatial transcriptomics in the 2010s [6–8]. In parallel, advances in mass spectrometry (MS) and mass spectrometry imaging (MSI) have produced label-free maps of protein localization (“spatial proteomics”) and, soon after, spatial metabolomics and lipidomics. Owing to the rapid maturation of these methods, Nature Methods selected spatial transcriptomics (2020) and spatial proteomics (2024) as the Methods of the Year, highlighting a broad revolution toward multiscale, multimolecular cartography [9, 10].

Spatial omics studies can be conducted via in situ or ex situ methods, depending on the platform; some in situ methods require microscopy, and some ex situ methods exploit high-throughput NGS, as MS can be applied for both [11]. MS thus serves as the Rosetta Stone of spatial omics for its multiomics versatility and ability to profile peptides, intact proteins, metabolites, lipids, glycans, and posttranslational modifications without prior knowledge of the targets on a single platform, which is unmatched by optical or sequencing-based approaches.

**Fig. 1:**
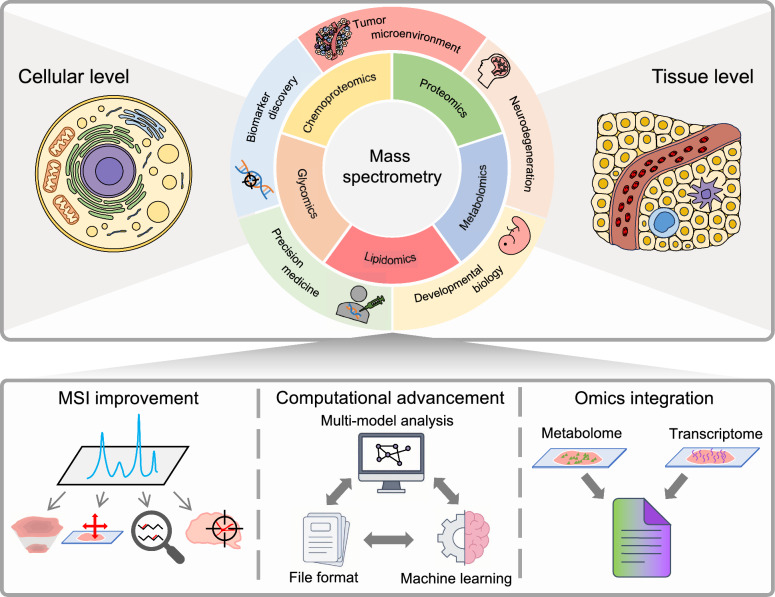
An overview of mass spectrometry-based methods and applications. Mass spectrometry-based methods, including proteomics, metabolomics, lipidomics, glycomics, and chemoproteomics, have been utilized to study the tumor microenvironment, neurodegenerative diseases, developmental biology, biomarker discovery, and precision medicine. Recent innovations in mass spectrometry technologies, analytical processes, and data integration provide deeper, more comprehensive insights into spatial omics

This review focuses on MS-based spatial omics technologies, highlighting their fundamentals, technical and computational innovations, and applications (Fig. 1). It first outlines the fundamental principles of MS and the four methodological quadrants of in situ versus ex situ and labeling versus label-free approaches. It then surveys key MSI platforms along with recent innovations for higher spatial resolution, broader and deeper molecular coverage, and faster data acquisition. Next, our attention turns to computational advancement, where cloud-optimized file formats, machine-learning-driven analysis pipelines, and cross-modality registration techniques collectively accelerate the processing and interpretation of large, multimodal datasets. Afterward, the discussion extends to strategies for the integration of MSI with other spatial omics modalities, demonstrating how complementary molecular maps unlock new biological insights. Afterward, the latest applications spanning the tumor microenvironment (TME), neurodegenerative disorders, developmental biology, biomarker discovery, and precision medicine highlight the clinical potential of MS-based spatial omics. Finally, the review discusses current obstacles and new opportunities, providing a comprehensive perspective on the rapidly changing technology landscape and its future clinical impact.

## Mass spectrometry fundamentals in spatial analysis

### Basic principles of mass spectrometry

To investigate the spatial distribution of molecules in the regions of interest, it is common to label and then observe them under microscopy. However, the label-based method requires prior knowledge about the molecules of interest and thus is not applicable for the investigation of unknown features, such as new molecules and drug metabolites and proteins with posttranslational modification. MS technologies are capable of high-throughput identification of proteins and metabolites without any labeling and thus complement label-based methods in unknown or highly complex biological samples.

**Fig. 2:**
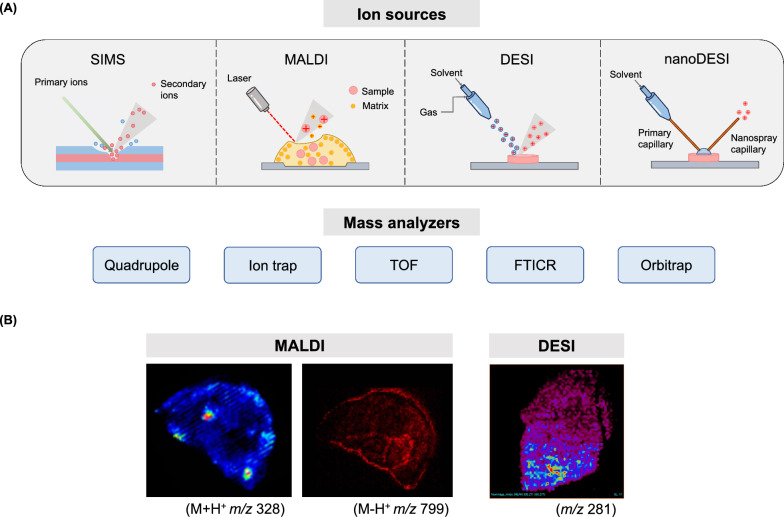
Types of ion source and mass analyzer of a mass spectrometer and representative MSI images. **A** A mass spectrometer is composed of three parts: an ion source, a mass analyzer, and a detector. Analytes are ionized by an ion source, separated by a mass analyzer, and captured by a detector to obtain the mass spectrum. The ion sources used for spatial omics include secondary ion mass spectrometry (SIMS), matrix-assisted laser desorption/ionization (MALDI), desorption electrospray ionization (DESI), and nano-DESI. Quadrupole, ion trap, time-of-flight (TOF), Fourier transform ion cyclotron resonance (FTICR), and Orbitrap are common mass analyzers. **B** Representative MALDI- and DESI-MSI images generated in our laboratory are shown as illustrative examples of spatial molecular distributions obtained from tissue sections, highlighting the distinct spatial patterns arising from laser-based and ambient ionization strategies. Animals using in our laboratory were approved by the Institutional Animal Care and Use Committee (IACUC) at National Taiwan University (Taipei, Taiwan)

A mass spectrometer (Fig. 2A) determines the mass of a molecule by measuring the mass-to-charge ratio (m/z) of its ion and is composed of three parts: an ion source, a mass analyzer, and a detector. Ions are generated by gaining positive or negative charges, and then sent to the mass analyzer for separation on the basis of their m/z for detection. The final output of the detector is a mass spectrum that reflects both the mass-to-charge ratios and the relative abundances of the detected ions. Ion sources convert neutral molecules into gas-phase ions, and different ionization mechanisms strongly influence desorption physics, ion yields, spatial resolution, and molecular coverage. Secondary ion mass spectrometry (SIMS), applied to biological analysis since the mid-1970s, uses a focused primary ion beam under high-vacuum conditions to sputter secondary ions from the tissue surface [12]. SIMS offers submicrometer spatial resolution, but its high-energy sputtering generally breaks down larger biomolecules, restricting detection primarily to elements, lipids, and small metabolites [13, 14]. Laser desorption/ionization (LDI) methods enabled photon-induced desorption in vacuum instruments, providing a comparatively less energetic and more controllable desorption environment than SIMS. Early implementations of LDI, however, still ionized mainly small molecules because larger biomolecules underwent extensive fragmentation. A major breakthrough came with matrix-assisted LDI (MALDI), where an energy-absorbing organic matrix mediates soft desorption and ionization of large biomolecules [15]. Around the same time, Tanaka and colleagues showed that metal-glycerol matrices could extend MALDI to high-molecular-weight analytes up to approximately 100 kDa, establishing MALDI as a core platform for protein and proteoform analysis [16]. MALDI was further developed for spatial tissue imaging, with Caprioli and colleagues demonstrating direct localization of peptides and proteins from tissue sections [17, 18]. MALDI continues to evolve through higher-resolution optics and laser post-ionization approaches such as MALDI-2, which enhances ion yields for lipids and metabolites by up to two orders of magnitude [19]. Beyond improvements in ionization efficiency, subsequent advances in matrix chemistry, sample preparation, and instrumental optimization have further expanded the accessible mass range of MALDI-based MSI. Notably, MALDI–TOF has been demonstrated to image intact proteins approaching 200 kDa directly from tissue sections, underscoring the continued relevance of TOF analyzers for high-mass protein imaging in spatial proteomics [20]. While MALDI-based approaches have continued to advance under vacuum conditions, ambient ionization sources extend MS to atmospheric-pressure environments. Desorption electrospray ionization (DESI) enables direct tissue analysis through charged-droplet impact without the need for vacuum or extensive sample preparation, making it compatible with hydrated or minimally processed samples [21]. DESI has been applied to spatial metabolomics and lipidomics, whereas protein and proteoform imaging by DESI remains less common and typically requires additional methodological considerations [22]. Because laser-based and ambient ionization sources sample tissues through different physical mechanisms, they often generate distinct spatial ion intensity patterns in practice; representative MALDI-MSI and DESI-MSI datasets are shown in Fig. 2B to illustrate typical spatial molecular distributions obtained using these complementary ionization strategies and to bridge schematic descriptions of instrumentation with real MSI data. Nanospray DESI (nano-DESI) modifies DESI by forming a stable microscale liquid junction between two capillaries, enabling more controlled and gentle analyte extraction from tissue surfaces and thus improving spatial precision and preserving noncovalent protein complexes. Nano-DESI has been applied from lipid and metabolite imaging to protein and proteoform-level analyses in tissues. Early studies established ambient tissue imaging using nano-DESI and demonstrated automated, high-resolution platforms suitable for quantitative phospholipid imaging [23–25]. More recently, nano-DESI has enabled label-free protein imaging and native mapping of protein complexes and proteoforms directly from tissue sections [26–28]. Together, these developments position nano-DESI as a complementary approach to MALDI in spatial proteomics, particularly when gentle sampling and native-like conditions are desired.

Following ionization, mass analyzers separate ions according to their mass-to-charge (m/z) ratios, and the choice of analyzer strongly affects mass range, resolving power, acquisition speed, and suitability for spatial imaging. Time-of-flight (TOF) analyzers are widely used in MSI because they are compatible with pulsed ionization sources such as MALDI, providing broad mass ranges and enabling rapid parallel detection which make them suitable for high-throughput tissue imaging of metabolites, lipids, and peptides [17, 29]. Fourier-transform-based analyzers, including Fourier-transform ion cyclotron resonance (FTICR) and Orbitrap, detect image currents generated by coherent ion motion and therefore offer ultrahigh mass resolving power and mass accuracy. When coupled with MALDI or nano-DESI ion sources, FTICR enables isotopic fine-structure resolution and intact protein or proteoform detection directly from tissue sections, including the discrimination of post-translational modifications and charge-state distributions, but at the cost of increased instrumental complexity and slower acquisition speeds [30–32]. Recent instrumental advances, including operation under reduced vacuum conditions, have further extended the accessible mass range of FTICR-based MSI for intact protein analysis [33]. Orbitrap analyzers provide a complementary balance between high resolution, robustness, and acquisition speed, and have been increasingly adopted in MSI workflows. In particular, MALDI- and nano-DESI-coupled Orbitrap platforms have enabled high-resolution spatial lipidomics and metabolomics, as well as emerging proteoform-resolved protein imaging directly from tissues [34–36]. Accordingly, the selection of a mass analyzer in spatial MS is inherently application-driven, reflecting trade-offs between mass range, resolving power, acquisition speed, and the level of structural information required—from high-throughput molecular mapping to posttranslational-modification- and proteoform-resolved imaging.

The final part of a mass spectrometer is the detector, which converts ion currents into measurable electrical signals [37]. In the context of spatial mass spectrometry, detector performance primarily influences sensitivity, dynamic range, and acquisition speed rather than molecular selectivity. Because most modern MSI platforms rely on mature and well-established detector technologies that are tightly integrated with the mass analyzer, detector design is generally not the limiting factor for spatial resolution or molecular coverage. Instead, ionization efficiency and mass analysis remain the dominant determinants of MSI performance.

From a system-level perspective, an ideal MS exhibits analytical attributes such as high ion detection efficiency, low or no noise, simultaneous detection, wide mass-range and mass-independent response, wide dynamic range, fast response and short recovery time, high signal saturation level and signal stability, and favorable operational attributes such as long life, low maintenance, ease of replacement, and low replacement cost [38]. To apply MS in spatial analysis, additional technological considerations should be incorporated, such as ionization in situ to retain spatial information and the trade-off between spatial resolution and analytic time.

### Main MS technologies for spatial applications

**Fig. 3:**
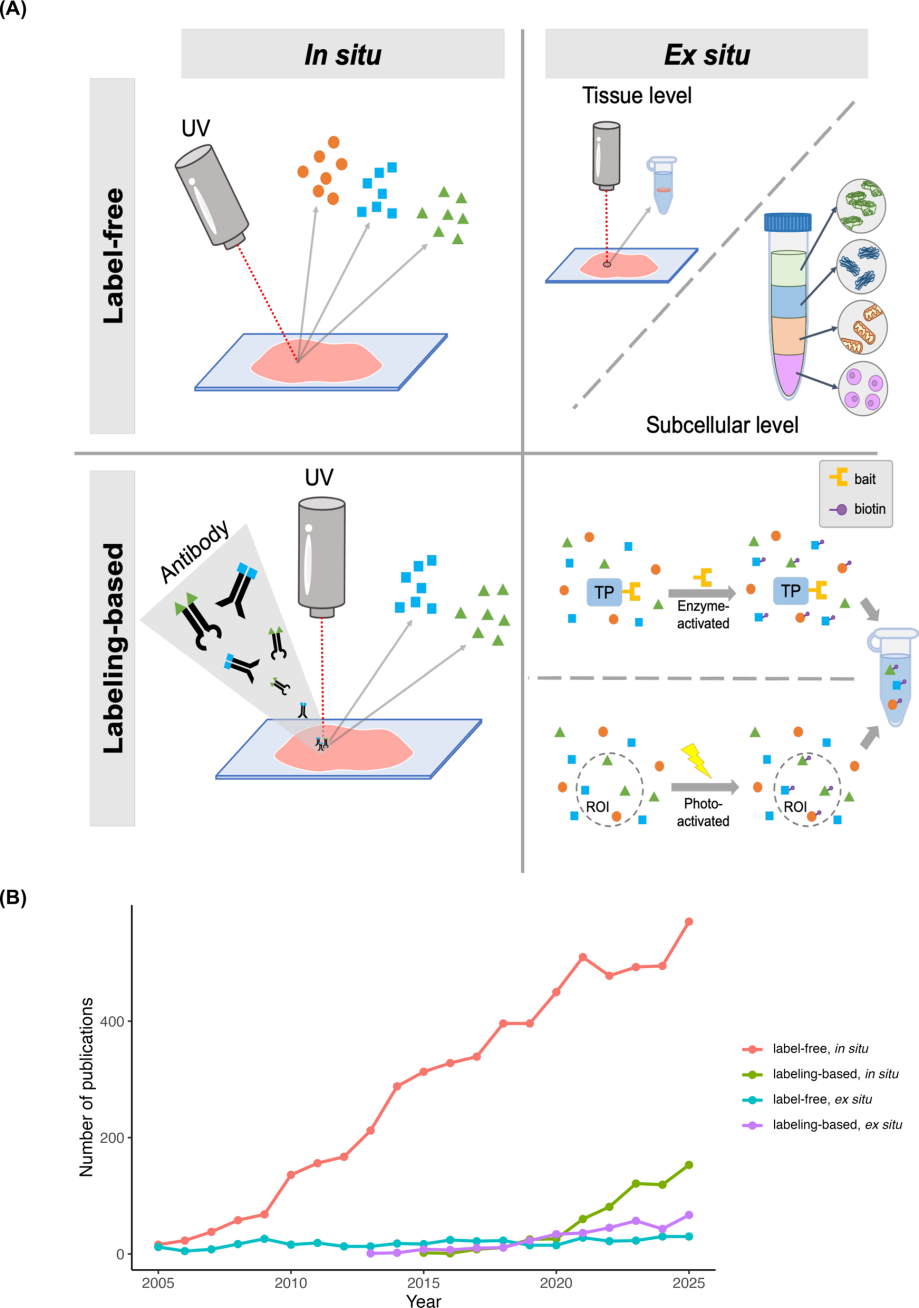
Main MS-based approaches. **A** MS-based technologies based on the analysis of place and labeling can be classified into four categories: mass spectrometry imaging (label-free, in situ), isotope/metal labeling (labeling-based, in situ), laser microdissection at the tissue scale and subcellular proteomics at the subcellular scale (label-free, ex situ), and proximity labeling (labeling-based, ex situ). (TP: target protein) **B** Publication trends of MS-based spatial omic approaches over two decades (2005–2025), categorized by major methodological classes. Publication counts were obtained from PubMed (December 31, 2025). Review articles were excluded because they may cover multiple technologies and could lead to repeated counting. Articles containing any of the following keywords in title or abstract—“mass spectrometry imaging”, “imaging mass spectrometry”, “MALDI-MSI “, “MALDI MSI”, “MALDI imaging”, “DESI imaging”, “DESI MSI”, “DESI-MSI”, “SIMS imaging”, “SIMS MSI”, or “SIMS-MSI”—were classified as label-free, in situ methods (MSI). Articles containing any of the following keywords in title and abstract—“imaging mass cytometry”, “multiplexed ion beam imaging”, “MIBI-TOF”, “MALDI-IHC”, “MALDI immunohistochemistry”, or “MALDI-ISH”—were classified as labeling-based, in situ methods (IMC/MIBI/MALDI-IHC/MALDI-ISH). Articles containing “mass spectrometry” together with either “laser capture microdissection" or “laser-capture microdissection” in the title or abstract were classified as label-free, ex situ methods (LCM-MS). Articles containing “mass spectrometry” and “proximity” together with any of the following keywords in title and abstract—“APEX”, “APEX2”, “BioID”, or “TurboID”—were classified as labeling-based, ex situ method (proximity labeling). Overall, these trends illustrate the dominant and continuously expanding role of label-free, in situ MSI in the spatial omics field

On the basis of the location of analysis and the use of labeling, MS-based omics methods can be roughly separated into four categories: (1) in situ and label-free methods, such as MSI; (2) in situ and labeling-based methods, such as imaging mass cytometry (IMC), multiplexed ion beam imaging (MIBI), and MALDI-immunohistochemistry (MALDI-IHC); (3) ex situ and label-free methods, which are predominantly based on laser capture microdissection (LCM) coupled with liquid chromatography–mass spectrometry (LC–MS) workflows; and (4) ex situ and labeling-based methods, such as proximity labeling (Table 1 and Fig. 3A) [39–56]. Although many MS-based spatial technologies were initially developed for proteomics, several of them are now widely applied to other molecular classes. For example, MSI is routinely used for metabolite, lipid, and glycan imaging, and LCM–MS workflows can be coupled with lipidomics or metabolomics [57, 58]. Accordingly, the four categories described below should be viewed as spatial MS modalities that operate across multiple molecular classes rather than proteomics-specific technologies.

**Table 1:** Key technologies of MS-based spatial omics

Technology	Location	Labeling	Superiority	Recommended scenarios	Methods	Resolution
MSI	In situ	No	Whole-slide coverage Unbiased discovery	Discovery research Multimodal integration	SIMS and derivatives [39] DESI-MSI and derivatives [40] MALDI-MSI and derivatives [41]	10–50 nm 150–200 μm 1–200 μm
Isotope/metal/photocleavable labeling	In situ	Yes	FFPE-friendly Subcellular resolution	High-throughput quantification Clinical validation	IMC [42] MIBI [43] MALDI-IHC [44] MALDI-ISH [59]	1 µm 260 nm 10 μm 20 μm
Laser microdissection	Ex situ	No	Morphology-guided Deep coverage	Cancer heterogeneity Drug discovery	LCM-MS [46] DVP [47]	Regional tissue Single cell
Subcellular proteomics	Ex situ	No	Proteome-wide organelle assignment Dynamic re-localization mapping	Subcellular map Molecular re-localization	LOPIT and derivatives [48, 49] DOMs [50]	Organelle Organelle
Proximity labeling	Ex situ	Yes	Nanometer-scale Real-time targeting	Transient interaction Microenvironmental interactome	APEX and derivatives [51, 52] BioID and derivatives [53, 54] Optoproteomics [55, 56]	20 nm 10–15 nm 240 nm

MSI methods scan the sectioned samples directly to generate ionized molecules, which are then sent to MS for molecular identification [39–41]. MSI is advantageous because it retains the spatial information and requires no prior knowledge of molecules; thus, it allows researchers to discover novel spatial patterns of molecules. In addition, multiple types of molecules can be detected in the same scan. However, MSI has limitations in terms of spatial resolution (generally at the μm level) and sensitivity for identifying low-abundance molecules. Therefore, MSI is recommended for quickly depicting comprehensive spatial patterns of molecules. In contrast, IMC and MIBI start with the use of metal-labeled antibodies to mark specific proteins on the sectioning samples, followed by MS detection of the metals that are ionized by laser (in IMC) or by ion beams (in MIBI) [42, 43]. Unlike MSI, IMC and MIBI use designed antibodies, thus requiring prior knowledge of protein targets. Although IMC and MIBI are not designed for unbiased discovery of novel or unexpected proteins, they are well suited for targeted biomarker detection owing to their high specificity, subcellular-scale spatial resolution (on the order of nanometers), and strong multiplexing capability, enabling the simultaneous imaging of more than 30 protein markers in situ. Complementing these metal-tag-based approaches, affinity-based labeling strategies employing photocleavable mass tags have recently broadened the scope of labeling-based spatial MS beyond IMC and MIBI. An emerging example is MALDI-IHC, which enables antibody-guided spatial proteomic profiling using mass-encoded reporters [44]. These affinity reagents, including commercially developed platforms such as Ambergen mass tags, enable highly multiplexed protein imaging using conventional MALDI instrumentation, without the need for specialized ion-beam systems. Recent studies have demonstrated sensitive and scalable MALDI-IHC workflows in FFPE tissues, positioning this approach as a practical bridge between conventional immunohistochemistry and MS-based spatial proteomics [45]. Moreover, the same photocleavable mass-tag concept has been extended beyond protein targets toward nucleic-acid-directed assays, including MALDI-based in situ hybridization (MALDI-ISH) strategies for spatial mRNA detection, further broadening the scope of affinity-based spatial MS [59].

At the tissue or cellular level, LCM–LC–MS workflows excise regions of interest (ROIs) from tissue sections and subjected to deep proteomic analysis. A range of implementations have been developed within this general framework, including classical image-guided LCM–LC–MS pipelines and more recent single-cell-resolved LCM-based workflows exemplified by deep visual proteomics [46, 47]. Recent technical advances have markedly enhanced both the depth and spatial resolution of LCM-based spatial proteomics, including nanodroplet processing in one pot for trace samples (nanoPOTS)-based ultrasensitive sample preparation, deep-ultraviolet laser ablation for subcellular protein sampling, and emerging one-pot proteomic workflows that reduce sample loss and handling complexity [60–62]. Together, these approaches enable high-coverage proteomic profiling from microscale or even subcellular regions; however, their broader adoption remains constrained by technical complexity and specialized instrumentation. At the subcellular level, fractionation-based methods such as localization of organelle proteins by isotope tagging (LOPIT), hyperLOPIT, and dynamic organelle maps (DOMs) can infer protein localization on the basis of abundance profiles across fractions, enabling high-resolution maps of protein colocalization and organelle-to-organelle translocation under different biological conditions [48–50]. Complementary to these fractionation methods, proximity labeling techniques (e.g., APEX, APEX2, BioID, and TurboID) identify proteins within a nanometer-scale radius of a protein of interest (the “bait” protein), enabling the characterization of highly localized proteomes with suborganelle resolution [51–54, 63]. A recent variant, termed “optoproteomics”, eliminates the need for a bait and instead uses image-guided photochemistry to label proteins within user-defined subcellular structures even in fixed or formalin-fixed paraffin-embedded (FFPE) tissue, allowing unbiased ROI-specific proteome profiling [55, 56].

Each of these methods is suited to different applications and has distinct limitations (Table 1). Considering the sample preparation requirements, operational costs, and the level of technical expertise required, MSI represents the most accessible and wide adopted entry point for spatial omics studies in general laboratories. This preference is reflected in the recent literature. As summarized in Fig. 3B, MSI-related publications have increased steadily over the past two decades and substantially outnumber those of other MS-based spatial approaches, including labeling-based imaging methods, LCM-based workflows, and proximity-labeling strategies. This sustained growth highlights the central role of MSI within the broader spatial omics landscape. Accordingly, the following sections focus on recent advancements in MSI technologies and analytic methods.

## Mass spectrometry imaging (MSI) technologies and advancements

**Fig. 4:**
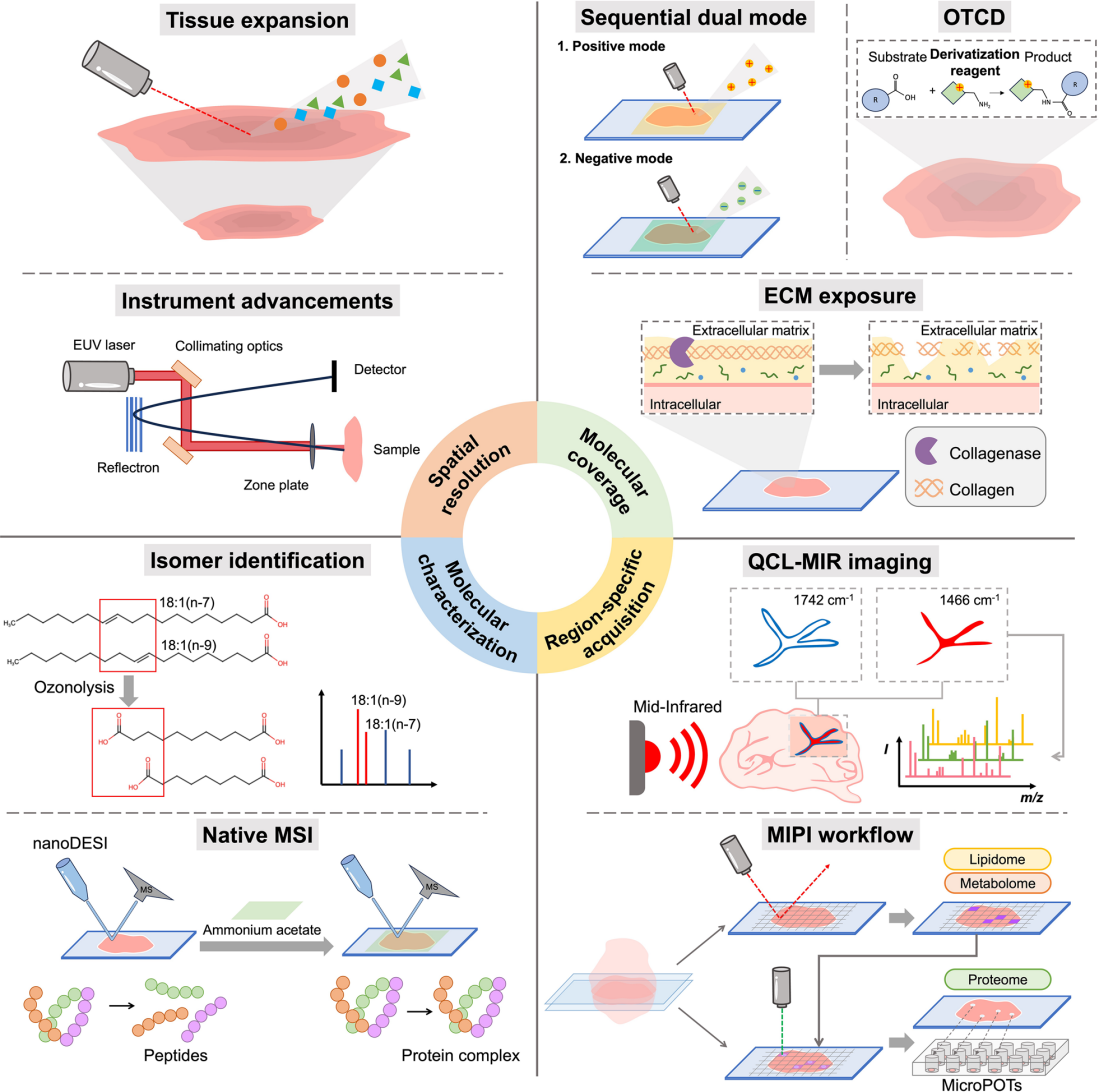
Recent technical advancements in MSI. The advancements are categorized into four major areas: (1) spatial resolution improvements via advanced instrumentation, tissue expansion techniques, and specialized treatment of hard tissues; (2) molecular coverage enhancement through increased ion yield and targeted acquisition strategies; (3) molecular characterization by isomer differentiation and preservation of native protein states; and (4) region-specific acquisition enabled by guided MSI using prior optical imaging or followed by high-resolution tandem MS

MSI enables label‑free mapping of hundreds of molecules across tissue slices. Among the various ionization techniques, MALDI, SIMS, DESI, and nano-DESI are the most commonly used ionization tools in MSI for biological samples because of their soft ionization capability, spatial resolution, and suitability for biological analysis. Recent advances in MSI have focused on four key themes (Fig. 4) [39–41]. First, spatial resolution has improved from tens of micrometers down to submicrometer and even nanometer scales. Second, molecular coverage has expanded, enabling the detection of a wide range of small metabolites within a single experiment and enhancing the visibility of rare or low-abundance species. Third, molecular characterization techniques now reveal detailed structures such as lipid double‑bond positions and native protein assemblies. Fourth, targeted acquisition strategies use prior optical imaging or broad MSI surveys to define ROIs before detailed acquisition, cutting instrument time and cost. Here, we review the latest developments in each of these areas, highlighting innovations that are reshaping the capabilities and applications of MSI.

### Spatial resolution

Spatial resolution in MSI has historically been constrained by ionization methods and sample preparation. In routine practice, commercially available MALDI MSI platforms typically achieve pixel sizes of approximately 5 µm, enabling single-cell-level spatial analysis. In addition, specialized MALDI ion sources have demonstrated submicrometer spatial resolution, with commercially available systems now capable of imaging below 1 µm [64]. Rather than uniformly improving resolution across all modalities, recent methodological developments have expanded the practical resolution range of MSI, pushing the lower bound to the subcellular scale while also enabling robust performance at larger pixel sizes for challenging or heterogeneous tissues. This broader operating range allows MSI to accommodate different analytical contexts, from high-resolution molecular mapping to large-area or structurally complex specimens, through three principal strategies.

First, instrument‑based advances have reduced pixel sizes. Integrated microfluidic probe designs have enabled for nano-DESI to reach spatial resolutions of approximately 8–10 µm through optimized channel geometry and stabilized liquid‑bridge designs [65]. In parallel, transmission‑mode MALDI combined with MALDI‑2 post‑ionization has enabled subcellular images with effective pixel sizes as small as 600 nm [66]. Furthermore, MALDI-2-enabled oversampling combines stage steps smaller than the laser spot to reduce effective pixel sizes to 6–8 µm with signal intensities comparable to those of conventional 20 µm scans [67]. Most MSI studies in this area employ deep ultraviolet (DUV) lasers (e.g., 153 nm or 193 nm excimer sources); however, exploratory work using extreme ultraviolet (EUV) laser ablation has been demonstrated to achieve lateral resolutions of approximately 75 nm and depth resolutions of approximately 20 nm in TOF MSI [68]. Second, tissue‑expansion methods such as gel‑assisted MSI (GAMSI) and tissue‑expansion MSI (TEMI) can be used to swell samples approximately 3–6-fold (and up to 10-fold for TEMI), allowing standard MALDI instruments to image at submicrometer pixel sizes without changing hardware [69, 70]. Third, for hard or uneven tissues, samples are freeze-dried, cut into 5 µm slices, and gently flattened by contactless spinning to prevent cracks and keep surfaces flat—this approach supports up to 10 µm resolution in challenging samples such as bone and cartilage [71]. Together, these strategies now enable the detectable range of MSI from large tissue regions down to single cells and nanovolume domains.

### Molecular coverage

Molecular coverage in MSI, the breadth and depth of detectable species, is often compromised by ion‑charge effects and analyte selectivity. To overcome these limitations, two complementary strategies have been developed. Universal methods increase overall ion detection without focusing on specific analytes. For example, sequential dual‑mode MALDI workflows with 1,5-diaminonaphthalene (DAN) and its hydrochloric salt (DAN–HCl) matrices double the total number of metabolites detected in a single experiment; notably, the dual polarity of 1,5-DAN matrix has also been demonstrated for lipid analysis [72, 73]. More recently, vacuum-stable, caged derivatives of dihydroxyacetophenone (DHAP) have been introduced as alternative dual-polarity MALDI matrices, offering comparable ionization performance while addressing toxicity and handling concerns associated with DAN [74]. The use of a low‑current O₂⁺ auxiliary beam improves positive‑ and negative‑ion yields on insulating samples, and sodium doping during gelatin-coated indium tin oxide (ITO) slide mounting separates isobaric and isomeric lipids in oat tissues [75, 76]. In contrast, targeted approaches focus on particular classes of molecules. A simple hexane wash removes abundant lipids and uncovers more than 50 regions of specific polar and ^2^H‑labeled metabolites in mouse organs [77]. Similar solvent-based washing and interference-removal strategies have been broadly applied to enhance metabolite coverage in MSI, including ammonium (NH₄⁺) rinses to modulate adduct formation and the reduction or removal of polyethylene glycol (PEG) to suppress polymer-derived background [78–80].

Targeted derivatization strategies further extend molecular coverage toward specific functional groups, such as carboxyl- and aldehyde-containing metabolites, by improving ionization efficiency and detection sensitivity [81, 82]. These approaches are collectively referred to as on-tissue chemical derivatization (OTCD), in which derivatization reagents such as tris(2-pyridylmethyl)amine (TMPA), in combination with peptide coupling agents, such as hexafluorophosphate azabenzotriazole tetramethyl uronium (HATU), are applied directly onto tissue sections to derivatize poorly ionizable or volatile metabolites, including short-chain fatty acids [83]. In parallel, enzymatic on-tissue treatments such as collagenase digestion have been used to enrich extracellular matrix (ECM) peptides, thereby improving the spatial imaging of ECM components [84]. Targeted strategies have also been developed to enhance protein and peptide coverage by controlling ion chemistry via acidification. Recent studies have demonstrated that controlled acidification markedly enhances protein and peptide MSI without extensive chemical derivatization, enabling more sensitive and selective protein imaging in tissues [36, 85]. Beyond chemical selectivity, instrumental innovations further extend targeted coverage [86]. Trapped ion mobility coupled with dual‑polarity ionization profiles hundreds of lipid species at single-cell resolution [87]. Furthermore, the integration of transmission-geometry atmospheric-pressure MALDI (AP-MALDI) with plasma post-ionization, together with a lipid-preserving pre-staining workflow, enables targeted enhancement of ion yield and supports concurrent high-resolution imaging of lipids and nucleotides, thereby effectively expanding molecular coverage at the subcellular scale [88]. Together, these universal and targeted strategies broaden and deepen MSI coverage, from small metabolites to peptides and proteins.

### Molecular characterization

Despite powerful mass measurements, detailed molecular characterization in MSI remains challenging because of isomeric differentiation and fragile noncovalent protein assemblies.

Singlet oxygen labeling with tetrakis(4-carboxyphenyl)porphyrin (TCPP) in nano‑DESI solvent converts C double bond sites into hydroperoxides whose unique fragmentation reveals bond positions in a single imaging run [89]. In‑source ozonolysis within the MALDI source, OzMALDI, results in the formation of lipid ozonides from all unsaturated species and the rapid assignment of double‑bond positions without extending the analysis time [90]. In situ chondroitinase digestion enables spatially resolved separation of isomeric glycosaminoglycan (GAG) oligosaccharides [91]. Silver-based methods encode isomeric structure through Ag⁺-dependent fragmentation, enabling confident annotation of steroid and prostaglandin regioisomers in MSI without chemical derivatization [92, 93].

Native protein assembly imaging uses gentle ambient sampling to preserve noncovalent interactions. Under native MSI conditions, nano-DESI with an ammonium acetate buffer enabled direct, top-down mapping of folded proteins from tissue at sub‑10 µm spatial resolution while via top‑down MS, preserving native conformations and noncovalent protein complexes [27]. This approach was then applied to image 113 kDa aquaporin‑0 tetramers in the eye lens. Proteins were extracted from detergent micelles, and tetrameric and subunit masses were determined within their spatial context [94]. Native ambient MSI subsequently captured endogenous assemblies of up to approximately 145 kDa in the brain, kidney, and liver, as demonstrated by in situ stoichiometry mapping of large protein complexes [95]. Crucially, tandem MSI of the rat brain preserved protein–ligand and protein–metal interactions, verifying the coordination of GTPase–ligand complexes and metal‑bound enzymes [28]. Finally, a simple tissue‑washing protocol selectively removed soluble proteins while retaining integral β‑barrel and α‑helical membrane proteins in thin sections, thereby enabling imaging of membrane protein assemblies with intact spatial distributions [96]. Together, these native MSI innovations allow the characterization of molecular masses, bond positions, isomer identities, and higher‑order assembly states of proteins directly in tissue.

### Region-specific acquisition

Region-specific acquisition focuses analytical effort on predefined tissue regions to improve efficiency and minimize data redundancy. Two workflows, Fourier transform infrared microscopy (FTIR) and quantum cascade laser mid-infrared microscopy (QCL‑MIR), efficiently generate a label-free image to segment the tissue into molecularly distinct regions at 5–10 µm resolution [97, 98]. These ROIs are then co-registered to the MSI instrument for focused data acquisition, reducing full‑section data by more than 95% and ensuring precise mapping of structures such as glomeruli or tumor margins. Another workflow, metabolome-informed proteome imaging (MIPI), uses MALDI‑MS imaging to survey whole tissue at micrometer resolution and identify subregions where specific metabolic activities are enriched. These sub-ROIs are then excised from adjacent sections for microdroplet processing in one pot for trace samples (microPOTS)‑based proteome profiling, enabling ultrasensitive detection of region‑specific enzymes and reconstruction of active pathways [99, 100]. Recent advances have extended this concept to perform MSI-guided ROI selection and microPOTS-based proteomics directly on the same tissue section, including implementations based on adapted MALDI-MSI substrates as well as nondestructive DESI-MSI [101, 102]. By integrating these workflows, MSI experiments can dramatically reduce the acquisition time and data load while preserving analytical depth in the ROI.

Taken together, advances in spatial resolution, molecular coverage, molecular characterization, and region-specific acquisition have made MSI a powerful tool for detailed profiling of biological samples. Along with a recent co-registration strategy that integrates transmission-mode MALDI-2 MSI with in-source bright-field and fluorescence microscopy at submicron resolution, these improvements deliver finer images, detect a broader range of molecules, and shorten acquisition times, but they also produce larger, more complex datasets with high dimensionality and spectral variability [103]. To turn these rich data into meaningful biological insights, dedicated computational workflows are essential. In the next section, we review recent computational innovations that address these emerging MSI data analysis challenges.

## Computational innovations for MSI analysis

**Fig. 5:**
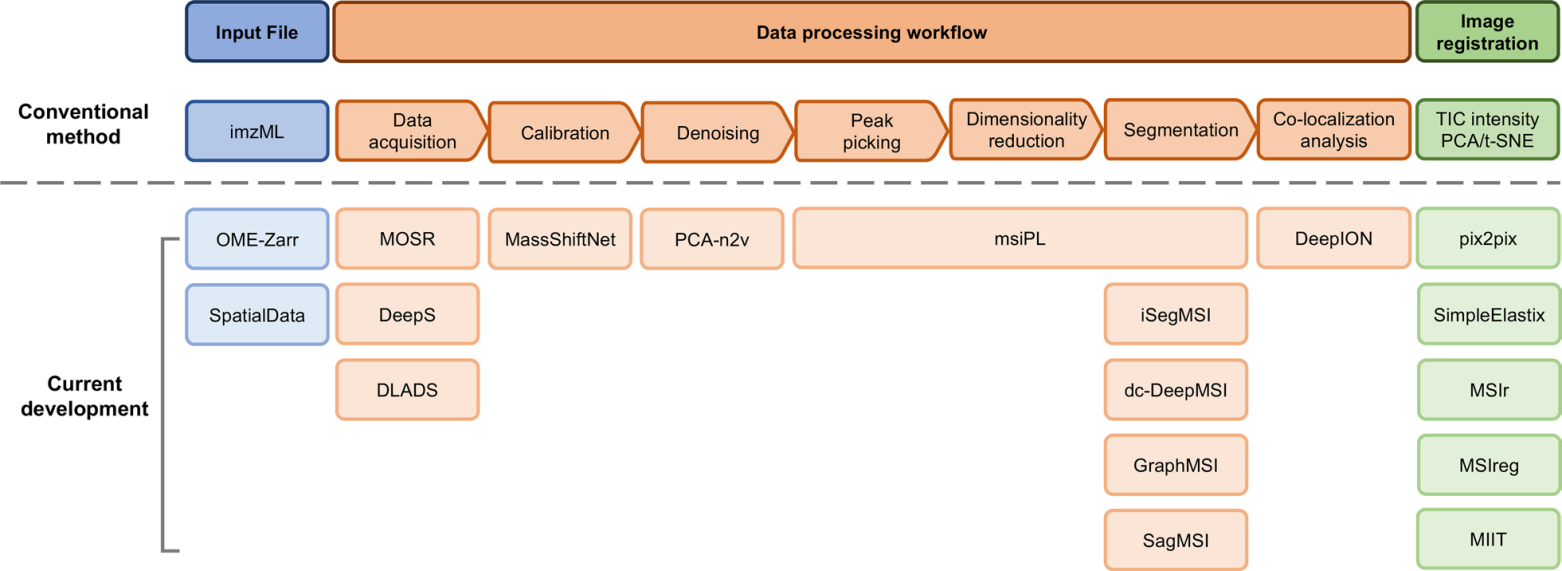
Computational innovations for MS-based omics. Three main improvements are summarized: (1) the input imzML file becomes a cloud-optimized format, (2) the data processing workflow incorporates machine learning methods, and (3) image registration is used for multimodal data

Currently, MSI can generate gigabytes or even terabytes of data. This expansion has created challenges for computational analysis, such as the storage and sharing of data, fast and accurate analysis of large-scale high-resolution spectra, and cross-modality registration that merges MSI with other spatial omics layers. Here, we review recent innovations that specifically address these challenges, focusing on file format advances, deep learning approaches embedded in the analytical pipeline, and improved algorithms that align MSI with complementary imaging modalities (Fig. 5). Rather than proposing a standardized workflow, Fig. 5 provides a conceptual overview of commonly used computational components, which are typically combined in different ways depending on the biological question, data modality, and laboratory practice.

### Cloud-optimized file format for big imaging data

Spatial omics methods generate multidimensional data, including position (x and y coordinates) and section (z coordinate), channels (markers for labeling methods or many m/z bins in MSI), ion intensity values (reflecting relative abundance), and time. A viable imaging format is expected to be a hierarchy of n-dimensional arrays with metadata so that each “chunk” of data, not the whole file, can be accessed. Current file formats for bioimaging, OME-TIFF and HDF5, are adopted for this purpose, but they have limitations in terms of data amount, complexity, and cloud-based storage [104, 105]. OME-TIFF is built of a five-dimensional data structure that is not designed for additional scientific axes such as m/z and thus is inefficient for MSI. HDF5 is more flexible in storing N-dimensional data but has no standardized attributes for describing image metadata or coordinate systems. To overcome these limitations, the OME-Zarr format, built upon Zarr, an open-source, chunked array storage format of large N-dimensional datasets, was developed for cloud-optimized data storage and is now widely adopted across many fields from genomics to astronomy and earth sciences [106, 107]. OME-Zarr enables remote handling of bioimage data, and its chunks can be streamed directly into downstream deep-learning pipelines. Afterward, an open and universal data framework for spatial omics, SpatialData, was developed [108]. It builds on five primitive elements (termed “SpatialElements”) for images, labels (e.g., segmentation labels), points (e.g., probe spots), shapes (e.g., ROI shapes), and tables (e.g., quantitative and annotation tables) and stores them as OME-Zarr chunks. Each dataset is registered into a common coordinate system (CCS) that is used to precisely locate points in space, enabling precise integration of datasets from diverse platforms, such as Visium, Xenium, and MIBI-TOF, on the same tissue section. It has a napari plugin, which is a viewer for multidimensional images in Python, for interactive spatial annotation and implements PyTorch to train deep learning models [109, 110]. In addition, it can analyze spatial data objects with analysis packages in the scverse ecosystem for single-cell and spatial omics data analysis, such as with Scanpy, Squidpy, and scvi tools [111–114]. Overall, SpatialData provides a unified file format, spatial coordinate framework, and analytical tools that simplify cross-platform and cross-study integration.

Currently, many OME-Zarr files are available in databases such as the BioImage Archive at EMBL-EBI and the Distributed Archives for Neurophysiology Data Integration (DANDI) of the NIH [115]. However, most of these datasets were derived from spatial transcriptomics platforms such as Visium and Xenium, as well as spatial proteomics datasets from IMC and MIBI-TOF. Although reports about OME-Zarr usage in MSI analysis are scarce, the common MSI data format, imzML, can now be converted to OME-Zarr through “pims-plugin-format-msi”, a plugin of the Python image management system (PIMS) of Cytomine [116]. New OME-Zarr specifications are anticipated to add an explicit m/z axis for the spectra, aligning the format with the high-dimensional needs of MSI workflows.

In parallel with emerging multimodal data frameworks, the MSI community has long relied on domain-specific data standards and repositories tailored to MS-based spatial data. The imzML/ibd format remains the primary open standard for MSI, supported by all major instrument vendors and widely used by analysis platforms such as METASPACE, which currently hosts more than 50,000 submitted MSI datasets spanning lipidomics, metabolomics, and spatial chemistry [117, 118]. METASPACE, together with the NIH Kidney Precision Medicine Project (KPMP) and the Human Biomolecular Atlas Program (HuBMAP), provides a major public infrastructure for MSI data storage, annotation, and reuse [119–121]. Notably, HuBMAP has additionally adopted OME-TIFF in combination with the Vitessce image viewer for multimodal visualization of MSI with other imaging modalities [122]. Together, METASPACE, KPMP, HuBMAP, and other NIH Common Fund programs form a federated ecosystem that supports both MSI-specific and multimodal spatial omics data, complementing emerging OME-Zarr-based frameworks rather than replacing established MSI standards.

### Analytical methods incorporating machine learning

Machine learning is a discipline devoted to building algorithms that learn from existing data to make predictions or decisions; like humans, their performance can be optimized as additional data are provided. Machine learning methods have also been applied in many fields of biology and medicine, especially those involving large-scale and high-dimensional data, such as those from omics and radiology. Because MSI yields omics-scale spectra and images with high spatial, chemical and biological complexity, dedicated machine learning modules have been introduced at almost every stage of the MSI workflow to help with data processing and interpretation.

In the data acquisition stage, deep learning approaches can be applied to increase spatial resolution while shortening scan times. A deep learning framework based on transfer learning, MSI from optical super-resolution (MOSR), transfers knowledge learned from abundant optical images and then fine-tunes it with ten high-spatial-resolution MSI datasets [123]. This technique decreases the demand for high-spatial-resolution MSI data for training and increases spatial resolution by tenfold, approaching the single-cell scale. From a complementary angle, DeepS targets three-dimensional (3D) MSI data [124]. It uses a 3D sparse sampling network (3D-SSNet) to reconstruct sparsely sampled tissue sections, and the image quality is the same as that of the conventional method, which uses full-sampling 3D MSI data even when 20–30% of the sections are sparse. A third strategy is to dynamically select the sparse informative pixels for data acquisition through the trained model to reduce the entire acquisition time and data amount. The deep learning approach for dynamic sampling (DLADS) employs a convolutional neural network to estimate the expected reduction in the distortion (ERD) of pixels, after which the algorithm selects pixels with high ERD for the next acquisition process [125]. DLADS was first applied for nano-DESI MSI with a single m/z channel and was expanded to multiple channels [126]. Later, it was implemented on a commercial mass spectrometer for nano-DESI MSI and was developed for MALDI‒MSI [127, 128]. DLADS can reduce the number of scanned pixels and scanning time by 40–80% with only a slight loss of image quality.

Instrumental biases, particularly matrix-induced drifts on MALDI platforms, are traditionally corrected with external or internal calibrants or by statistical schemes such as Kendrick mass-defect regression. A neural network-based method, MassShiftNet, provides a self-supervised learning framework for mass recalibration [129]. In the original article, MassShiftNet achieved a mass dispersion of 11.27 ppm, which is close to the expected misalignment of approximately 10 ppm under ideal conditions. For denoising, PCA-n2v first implements a principal component analysis (PCA) to decompose the MSI data into score images and then denoises these score images by a self-supervised convolutional-neural-network-based denoising method, Noise2Void (N2V) [130, 131]. PCA-n2v substantially increases the signal-to-noise ratio, revealing weak peaks that were previously buried in noise. Compared with the mature traditional methods, current deep learning-based methods offer better performance in terms of speed, analytical scale, and precision, which aligns with the rapid growth of MSI data.

In the traditional workflow (as shown in the center of Fig. 5), after instrumental errors have been corrected, peak picking, dimensionality reduction, and segmentation are fundamental for compressing the dataset while retaining the representative spectral features needed for downstream annotation and biomarker identification. Current deep learning approaches no longer follow the traditional linear workflow but instead implement an integrated, data-driven pipeline that collapses these linear steps into a single module. The peak-picking tool msiPL eliminates the subjectivity introduced by the selection of parameters during manual peak picking, such as baseline subtraction, peak width, signal-to-noise ratio, and smoothing [132]. It streams calibrated spectra directly to the implemented variational autoencoder (VAE), which performs nonlinear dimensionality reduction and spectral reconstruction, and automatically highlights the peaks that best explain spatial heterogeneity in the tissue, making segmentation an inherent part of this workflow. Beyond msiPL, several specialized modules were used for downstream analyses. Four segmentation methods, iSegMSI, dc-DeepMSI, GraphMSI, and SagMSI, are used to compartment heterogeneous ROIs by spectral similarity and spatial information while different neural networks are implemented [133–136]. Although iSegMSI still relies on preselected peaks and external knowledge for the boundary of heterogeneous ROIs, the other three methods are fully unsupervised methods without manual peak picking. After segmentation has been performed, the analytical focus shifts back to the ion-image layer, where representation learning and colocalization across thousands of ion channels become the primary goals. DeepION exactly addresses this process: It employs a SimSiam-based self-supervised contrastive learning model to compress tens of thousands of ion images into a 20-dimensional space, automatically clustering colocalized molecules and isotopic peaks [137]. By incorporating Poisson noise and missing value augmentation, DeepION preserves accuracy and GPU efficiency even for terabyte-scale MSI data with low signal-to-noise ratios.

Collectively, these advances illustrate how current deep learning methods can obtain high-quality MSI data while markedly reducing experimental effort and cost and can replace or outperform conventional calibration, denoising, peak picking, and segmentation methods, thereby streamlining the entire MSI analytical workflow. Beyond improving segmentation accuracy, recent work has increasingly focused on making MSI analysis results easier to understand and relate to underlying biology. For example, model-explainability approaches such as Shapley additive explanations (SHAP) have been used as post hoc tools to examine MSI segmentation or classification models and identify which m/z features contribute most strongly to specific tissue regions [138]. Although these methods are not part of a standardized MSI analysis pipeline, they help researchers interpret model outputs and assess whether region definitions are driven by biologically meaningful molecular signals. Along similar lines, image fusion approaches have been developed to combine MSI with complementary imaging modalities, such as histology or optical microscopy [139, 140]. Image fusion seeks to integrate the molecular specificity of MSI with the spatial detail and structural context provided by other imaging techniques, improving both visualization and interpretation. Early studies demonstrated multivariate and machine-learning-based fusion of MSI with histological images to enhance tissue classification and molecular localization, and more recent work has extended these strategies to ambient MSI platforms, including nano-DESI, enabling the integration of molecular information with optical or morphological features at cellular or subcellular scales.

### Cross-modality registration

Image registration aims to spatially align two or more images of the same biological specimen that were captured at different times and/or from different imaging modalities. Early developments focused on radiological data, such as magnetic resonance imaging (MRI), computed tomography (CT) scans, and ultrasound. These images are usually grayscale or contain only a few channels, and the signal intensity is usually linearly correlated with tissue density; thus, they are amenable to classical intensity-based registration. However, for MSI data, the situation is fundamentally different, as each pixel in the raster contains tens of thousands of m/z channels whose intensities report molecular abundance rather than morphology; that is, no simple monotonic relationship with tissue density exists. Moreover, spatial omics studies often combine MSI with histological hematoxylin and eosin (H&E) staining, immunofluorescence, or IMC on adjacent sections, thus requiring cross-modality registration that should also compensate for section-to-section distortions.

To make MSI data suitable for geometric alignment, the first step is to convert it to low-dimensional data that recapitulate gross morphology. One common method is to use the total ion current (TIC) of each pixel to represent intensity, as the TIC value is approximately proportional to the bulk molecular (and thus tissue) density. Another method involves performing dimensionality reduction by PCA or t-distributed stochastic neighbor embedding (t-SNE) and mapping the first three principal components (PCs) or t-SNE components to generate pseudo-RGB images [141, 142]. Recently, virtual histology images generated by pix2pix, a conditional generative adversarial network (cGAN) model, have been used to translate MSI data into synthetic H&E images [143]. Image registration is subsequently performed in two stages: First, a rigid or affine transform at the coarse scale removes global translation, rotation, scaling, and shear, and then a B-spline nonrigid transform at the fine scale refines the mapping by modeling local stretching. Finally, similarity is maximized with mutual information (MI) for cross-modal pairs or with normalized cross-correlation (NCC) when the gray-level correspondence is approximately linear.

The open-source package elastix implements this coarse-to-fine workflow, and its Python/R wrapper SimpleElastix allows users to run elastix in Python, Java, R, Octave, Ruby, Lua, Tcl, and C# on Linux, Mac, and Windows platforms through a few lines of code, making it an easy pipeline for image registration for large-scale, multiomics data [144, 145]. Recently, elastix-based registration has been further simplified for wider usage. MSIr is an automatic registration web service that wraps SimpleElastix, enabling users to upload their MSI files and H&E images to obtain the registered images [146]. An open-source R package, MSIreg, was developed on the basis of Cardinal for MSI data preprocessing, EBImage for H&E image preparation, and SimpleITK for the final registration and transformation [147–150]. It was designed for researchers who have no prior experience with image registration, reducing user input to a minimal set of parameters. These elastix-based tools are algorithmically mature with transparent parameterization, but they require manual choices of preprocessing and tend to converge to local optima when the paired modalities exhibit large elastic deformations or extreme differences in brightness, resolution, or field of view. Recently, a Python framework, Multi-Omics Imaging Integration Toolset (MIIT), was developed for integrating spatially resolved multiomics data with a registration algorithm, GreedyFHist, which was developed in the same study [151]. The GreedyFHist algorithm first extracts the tissue area based on the YOLOv8 object detection model and then implements the diffeomorphic registration tool Greedy for global affine registration, followed by local nonrigid registration. Compared with the conventional method, which requires extensive parameter tuning, MIIT provides an open-source, customizable pipeline that runs efficiently on CPUs with almost no parameter tuning, making it easy to run automatic batch registration and extend to any additional spatial omics modality.

In summary, the cloud-optimized file format, deep learning frameworks, and image registration workflows convert MSI from an isolated mass spectrometric technique into an interoperable member of the spatial omics field. We expect that these improvements will eliminate more technical barriers and support routine single-cell and even clinical MSI studies. Next, we introduce how MSI integrates with other spatial omics technologies.

## Integration with other spatial omics technologies

With the rapid advancement of spatial omics technologies, researchers can now investigate molecular signals from multiple modalities within the same spatial context. As the main approach of MS-based spatial omics, MSI has great strength in detecting thousands of molecules within a single section, and workflows specific for proteins, metabolites, or lipids have been developed. However, depicting the comprehensive mechanism of a tissue microenvironment with a single MSI dataset is difficult; thus, the integration of different spatial omics data is indispensable for understanding cellular heterogeneity. While several recent reviews have focused on specific aspects of MSI integration, we herein highlight recent advances that demonstrate how MSI can be practically combined with other spatial modalities within a unified analytical framework [152–154]. In this section, we discuss the applications and challenges of integration, focusing on (1) the acquisition of varied MSI-based omics data, (2) complementarities among different MS-based approaches, and (3) multimodal analysis with high-throughput data from non-MS platforms.

Although MSI technology can be applied to detect multiple molecular classes, including proteomic, metabolomic, lipidomic, and glycomic data, signals from different molecular types cannot be collected simultaneously under a single experimental setting; thus, adjacent tissue sections are commonly used to obtain integrable datasets [155, 156]. However, molecular identification from different sections may increase the error caused by image registration. Currently, some studies have demonstrated that, with appropriate sequencing of data acquisition or optimization of experimental conditions, multiomics MSI data can be obtained from a single tissue section. For example, sequential MALDI-MSI of lipids, N-glycans, and tryptic peptides on the same section highlights the enhanced molecular characterization achievable through integration of spatial multiomics analysis [157, 158]. In the DESI-MSI platform, a new workflow was developed to identify spatial metabolite patterns prior to spatial proteomic analyses of the same tissue section [102]. Although these methods provide precise integration of multiomics information, future optimization of experimental parameters and detection order is needed to maximize sensitivity and minimize signal loss across diverse tissues and molecular types.

While MSI methods generate wide-coverage molecular maps, labeling-based MS techniques such as IMC and MIBI quantify tens of preselected protein targets. Integrating MSI with IMC/MIBI thus enables untargeted discovery together with target-specific validation. Owing to their high multiplexing capacity and subcellular resolution, IMC and MIBI are widely applied to characterize and understand the TME for new therapeutic target discovery. Therefore, their integration with MSI is particularly valuable for revealing the highly heterogeneous complexity of the cellular composition and metabolic landscape of the TME. However, it is still challenging to precisely integrate these multimodal data. First, as the resolution of the MSI data is lower than that of the IMC/MIBI data (Table 1), the IMC/MIBI data must be projected to the MSI coordinate system or vice versa to align the two types of data onto the same raster image. Second, data dimensionality and signal type differ greatly between MSI and IMC/MIBI data, where MSI produces high-dimensional continuous spectra and IMC/MIBI data are signals of one-dimensional counts of intensity, making it difficult to compare MSI data with IMC/MIBI data directly in a simple linear manner. Recently, two studies have demonstrated the integration of MALDI-MSI and IMC data, opening the door to comprehensive spatial multiomics profiling. Nunes et al. developed an experimental protocol and an analytical framework to integrate MSI-based metabolomics and IMC-based immunophenotyping on the same section, providing a powerful tool for linking cellular composition and metabolic phenotypes [159]. Similarly, a reproducible semiautomated workflow, mass imaging modality integration coregistration (MIMIC), was developed for analyzing MALDI-MSI data with IMC on the same or consecutive tissue sections [160]. Collectively, these approaches establish a practical benchmark for MSI−IMC integration and lay an experimental foundation for coupling additional labeling-based platforms to MSI.

As MSI methods capture functional signals of proteins, metabolites, and small molecules, spatial transcriptomics and epigenomics techniques yield high-throughput information on gene expression and regulatory states, thereby revealing cellular status and regulatory potential in a spatial context. Merging these two data types would yield a comprehensive gene–protein–metabolite map. Sun et al. used adjacent sections to perform spatial metabolomics and lipidomics with air-flow-assisted desorption electrospray ionization-mass spectrometry imaging (AFADESI-MSI) and MALDI-MSI, respectively, as well as spatial transcriptomics by Visium [161]. Vicari et al. developed a workflow that is compatible with commercially available platforms, requiring almost no modifications to Visium glass slides, MALDI-MSI, or spatial transcriptomics protocols [162]. Their work combined histology, MSI, and spatial transcriptomics, enabling the spatial profiling of metabolites and gene expression within a tissue section. Godfrey et al. presented a spatial metabolomics and transcriptomics workflow on the same tissue section by performing DESI‒MSI, H&E staining, and Visium analyses sequentially, preserving RNA quality and data integrity [163]. Furthermore, an R package, SpaMTP, was designed for the integrative analysis of spatial metabolomics and transcriptomics data, bridging the commonly used MSI analytical tool Cardinal for metabolomics with the widely used single-cell analytical tool Seurat for transcriptomics [164]. To date, all these workflows and tools have been implemented to combine metabolome-level MSI data with spatial transcriptomics, but tools for proteome-level MSI data are lacking, indicating that the integration of proteome-level MSI data with cross-modal omics data is still challenging. These current workflows provide the basis for extending the same strategy to incorporate proteome-level MSI to achieve truly comprehensive spatial multiomics.

In summary, recent advances demonstrate that MSI can be coregistered with high-resolution labeling-based imaging and integrated with spatial transcriptomics on the same or adjacent tissue sections. These complementary approaches collectively move the field from single-modality pictures toward a comprehensive, in situ multiomics map. The next step is to optimize data acquisition orders, registration algorithms, and cross-platform analysis so that MSI can be fused with other spatial layers, ultimately yielding an integrated tissue atlas that links the transcriptome, proteome, metabolome, and other types of omics data within the native microenvironment.

## Applications in human biology and medicine

MS-based spatial technologies enhance our understanding of human diseases and biology by providing spatial information, enabling scientists to characterize genes, proteins, and cells within the tissue environment. In this section, we summarize established applications of MS-based spatial technologies in human biology and medicine, with a primary focus on MSI, which remains the most widely adopted platform for tissue- and cell-level spatial analysis. Beyond MSI, the spatial proteomics landscape also includes several complementary approaches, such as LCM–MS workflows, subcellular fractionation methods (e.g., LOPIT, hyperLOPIT, DOMs), and proximity-labelling strategies, which resolve protein organization at cellular or subcellular scales. These modalities have been widely used to study protein localization, microdomain-specific responses, and mechanistic signaling processes in cell systems. However, because their current applications currently lie predominantly in cell-based or mechanistic studies rather than in human tissue imaging, they are not the primary focus of the disease-oriented applications summarized below.

### Characterization of the tumor microenvironment (TME)

The TME—a highly dynamic ecosystem of tumor cells, stromal components, and diverse immune populations—plays crucial roles in tumor progression, immune evasion and therapeutic response [165–167]. Studies of the TME help scientists elucidate the underlying interaction mechanism between malignant cells and nonmalignant cells [165]. Bulk and single-cell technologies provide information on cell composition, but spatial information is usually erased through tissue disaggregation [165, 166]. Additionally, traditional protein-based techniques such as IHC and immunofluorescence are limited to detecting a few targeted proteins [168]. These factors constrain the capacity to precisely characterize the TME. Spatial profiling technologies now bridge this gap by pinpointing cell locations, mapping biomarker gradients, and resolving cell–cell interactions in situ. Moreover, the subregions of the TME might lead to intratumor heterogeneity and different therapeutic outcomes. Spatial features, such as immune infiltration, drug response and immunotherapy response, are crucial for understanding the activity of these subregions [166]. These features provide new insights into the comprehensive understanding of the TME mechanism and more informative findings in diseases, indicating that revealing the complexity of the TME is promising [167]. Recent MS-based spatial omics methods help elucidate the TME mechanism in terms of proteomics, lipidomics, and metabolomics (Table 2). In neuroblastoma, MYCN amplification, as a subset of high-risk neuroblastoma, influences patient survival and tumor progression. Zhu et al. profiled spatial glycomics using MALDI-MSI and revealed that the MYCN amplification region has high core fucosylation activity, suggesting oncogene-driven metabolomic rewiring [169]. Another example can be found in a study of lung squamous cell carcinoma (LUSC); spatial metabolomic clusters defined by metabolic heterogeneity using MALDI-MSI and IHC revealed that metabolic heterogeneity is negatively correlated with CD3 + /CD8 + tumor-infiltrating T lymphocytes (TILs) but positively correlated with PD-L1 expression, indicating that nutrient competition is an immune suppressive mechanism in the TME [170]. Complementarily, Lagache et al. developed a “dry proteomics” workflow to integrate MSI-based spatial lipidomics and proteomics, revealing that tumor heterogeneity in glioblastoma regions is strongly correlated with necrosis, immune response, tumor invasion, and specific proteins and metabolic pathways such as seleno-amino acid metabolism and L1 cell adhesion molecule (L1CAM) interactions [171].

**Table 2:** Applications of MS-based spatial omics for the characterization of tumor microenvironment

MS method	Tumor type	Targets	Main findings	References
MALDI-MSI	Neuroblastoma	Glycome	MYCN amplification enhances the expression of core fucosylation enzymes, with N-MYC serving as a key regulator	Zhu et al. (2025) [169]
MALDI-MSI	LUSC	Metabolome	Metabolic heterogeneity is highly correlated with immune suppression via nutrient competition with CD3 + /CD8 + TILs	Wang et al. (2025) [170]
MALDI-MSI	Glioblastoma	Proteome, lipidome	Specific protein and metabolic pathways, including selenoamino acid metabolism and L1CAM interactions, are strongly associated with high tumor heterogeneity and impact patient prognosis	Lagache et al. (2025) [171]
MALDI-MSI	Lung cancer	Metabolome	A strong spatial correlation is observed between GUK1 phosphorylation and guanine nucleotides enrichment in ALK + lung cancer in situ	Schneider et al. (2025) [205]
MALDI-MSI	Melanoma	Proteome	SIRT1 functions as an upstream negative regulator of mTOR signaling and represents a therapeutically actionable target within the human melanoma tumor microenvironment	Placke et al. (2025) [206]
MIBI-TOF	Neuroblastoma	Proteome	The neuroblastoma tumor microenvironment exhibits a highly spatially heterogeneous and dynamic immune landscape	Patel et al. (2024) [207]
TOF–SIMS	Breast cancer	Lipidome	ToF–SIMS-based spatial lipidomics reveals treatment-induced chemical alterations in cancer cells, identifying lipid droplet accumulation as a potential resistance marker	Manaprasertsak et al. (2025) [208]

Collectively, these studies demonstrate that MS-based spatial omics methods uniquely capture the biochemical heterogeneity of the TME and its immunoregulatory circuits, offering mechanistic biomarkers and actionable targets. Future efforts should focus on increasing subcellular resolution, quantitative cross-modality integration, and prospective clinical validation to fully translate spatial proteo-metabolomics into precision oncology.

### Neurodegenerative disease (ND) studies

With the extension of the human lifespan, the incidence of NDs has also increased. These diseases typically affect brain function in elderly individuals, leading to progressive declines in cognitive and behavioral abilities. Epidemiological data suggest that aging is a major accelerating factor for the development of NDs. Several hypotheses regarding disease pathogenesis—such as the amyloid-beta (Aβ) hypothesis, the tau protein hypothesis, and the lipid and glucose metabolism dysfunction hypothesis—indicate that abnormal protein accumulation is closely associated with the onset of various NDs [172, 173]. Traditional IHC and ISH technologies face challenges in terms of chemical specificity, selectivity, and spatial resolution [174]. In contrast, MSI technologies offer high chemical specificity and high sensitivity for spatial omics, such as lipidomics, metabolomics, and proteomics, within single tissue sections (Table 3). Soft ionization techniques, including MALDI and ESI, play crucial roles in advancing proteomics by enabling the characterization of proteins and providing spatial information within the highly complex environment of the central nervous system (CNS), thereby helping delineate neuronal mechanisms [174]. Spatial proteomics has demonstrated considerable value for revealing the spatial distribution of pathogenic proteins and the molecular mechanism of complex diseases [175].

**Table 3:** Applications of MS-based spatial omics for neurogenerative disease studies

MS method	Disease type	Targets	Main findings	References
MALDI-MSI/LC–MS	Alzheimer’s disease	Proteome, lipidome	Mapped over 15 N- and C-terminal Aβ proteoforms in post-mortem Alzheimer’s brains, uncovering microtubule-related pathways involved in synaptic degeneration	Toyama et al. (2024) [176]
MIBI	Alzheimer’s disease	Proteome	Microglia in the hippocampal CA1 region of Alzheimer’s disease cases showed a strong immune activation bias	Mrdjen et al. (2025) [177]
NanoSIMS	Human induced pluripotent stem cell	Proteome	Distinct organelle-specific protein turnover rates, shaped by precursor amino acid composition, offer a quantitative benchmark for protein dynamics in healthy neural progenitor cells	Lork et al. (2024) [178]
MIBI-TOF	Alzheimer’s disease	Proteome	Microglia were observed interacting with pathological tau aggregates in the hippocampal CA1 region of AD dementia	Vijayaragavan et al. (2022) [209]

In Alzheimer’s disease (AD), Toyama et al. integrated MALDI-MSI with liquid chromatography–tandem mass spectrometry (LC‒MS/MS) to map more than 15 N‑ and C‑terminal Aβ proteoforms in postmortem Alzheimer’s brains, revealing microtubule‑related pathways involved in synaptic degeneration [176]. Complementary on-tissue tryptic MSI, receiver operating characteristic (ROC)-filtered shotgun proteomics, and MALDI lipid imaging revealed plaque-associated phosphatidylcholines and white matter microbleeds, highlighting region-specific biomarkers beyond Aβ and tau. Moreover, Mrdjen et al. used MIBI to profile major brain cell types, AD hallmark proteins and 17 microglial phenotypes in postmortem human brains from normal individuals and AD patients [177]. They applied microglial state continuum (MSC) immune activation to different local niches in the brain. Compared with those from cognitively normal individuals, microglia from AD patients demonstrated a strong bias toward immune activation, accompanied by reduced homeostatic functions, increased inflammation-associated proteins, and dysregulated phagocytic activity, most notably in the hippocampal CA1 region. Taken together, the results of this study provide a spatial framework for gaining deeper insights into microglial states across different brain regions in health and disease, offering new directions for potential therapeutic strategies.

At the cellular level, neural progenitor cells (NPCs) play a pivotal role in neurodegenerative diseases by contributing to the regeneration of damaged neural tissue through their ability to differentiate into various neural cell types. Lork et al. employed correlative transmission electron microscopy and nanoscale secondary-ion mass spectrometry (TEM-NanoSIMS) to perform a pulse-chase experiment using ^15^N-labeled amino acids, generating organelle-resolved protein turnover maps at ~ 50 nm resolution in single human induced pluripotent stem cell (hiPSC)-derived neural progenitor cells [178]. Their findings revealed that turnover kinetics differ substantially across organelles and are influenced by the identity of the precursor amino acid, thereby establishing a quantitative baseline for protein dynamics in healthy neural precursors. Because disrupted proteostasis is a hallmark of neurodegeneration, this single-organelle turnover platform can now be transferred to patient-specific or genetically engineered neural models to pinpoint where and when clearance pathways fail during disease progression, offering a powerful tool for mechanistic studies and therapeutic screening.

Collectively, MS-based spatial omics is transforming ND research by linking biochemical heterogeneity to anatomical loci. Future efforts must focus on single-cell, even suborganelle, quantitation and the integration of multimodal data with longitudinal clinical cohorts to translate spatial signatures into early diagnostics and mechanism-based therapeutics.

### Developmental biology insights

Developmental biology is the field of revealing the time series of developmental changes and mechanisms in cells, tissues, and organisms. Developmental gene regulation is correlated with disease [179]. Traditional proteomics and metabolomics techniques lack in situ resolution, which limits the ability to extract spatially resolved molecular information from tissues. In contrast, MS-based spatial omics directly captures cellular functions and states, offering a more precise understanding than genomics or transcriptomics does [180]. Spatial proteomics provides high-resolution localization of proteins, enabling the characterization of proteomic profiles across distinct tissue regions and offering deeper insights into developmental mechanisms at unprecedented spatial precision. Micrometer-scale, multimodal MS imaging now reveals compartment-specific enzyme landscapes within placental villi, highlighting the growing importance of spatial proteomics in developmental biology, as summarized in Table 4 [100].

**Table 4:** Applications of MS-based spatial omics for developmental biology

MS method	Cell type	Targets	Main findings	References
MALDI-MSI/LC–MS	Placental villous	Proteome, lipidome, metabolome	Metabolic heterogeneity between the two compartments of placental villi	Veličković et al. (2025) [100]
NanoSIMS	Human induced pluripotent stem cell	Proteome	Assessing protein turnover as a key regulatory mechanism during cell differentiation and the progression of organismal development	Lork et al. (2024) [178]
Single-cell mass spectrometry	Embryo	Proteome	Gps1 and Nedd8 expression differ significantly between alpha and beta blastomeres, influencing lineage segregation	Iwamoto-Stohl et al. (2024) [181]

In the study of Veličković et al., spatial lipidomics, metabolomics, and proteomics were integrated to reveal the metabolic heterogeneity between the two compartments of the placental villous, syncytiotrophoblast (STB) and villous core [100]. By combining high-resolution MALDI-MSI (including on-tissue chemical derivatization) with microPOTS-LC‒MS/MS proteomics, STB is enriched for lipid and steroid biosynthetic pathways, whereas the villous core is characterized by ketone body oxidation and extracellular matrix remodeling. Their molecular-level findings provide new evidence for understanding how placental functional zones cooperate during late gestational development through complementary metabolic roles. Moreover, Lork et al. highlights that correlative TEM-NanoSIMS, by quantifying organelle-resolved protein turnover in single human iPSC-derived neural progenitor cells, can be directly applied to developmental questions [178]. These authors noted its ability to monitor protein turnover during cell differentiation and organismal development and proposed a method as a foundation for dissecting subcellular mechanisms that steer stem cell fate decisions. Validation in postmitotic, differentiated progeny further demonstrated the platform’s relevance for probing proteome renewal across developmental transitions. In the study of Iwamoto-Stohl et al., 2-cell mouse and human embryos were shown to contain alpha and beta blastomeres, which are defined by distinct protein abundance profiles enriched for functions in protein synthesis, transport, and degradation [181]. The expression of proteins such as Gps1 and Nedd8 significantly differs between blastomeres and influences lineage segregation. Furthermore, observations revealed that beta blastomeres contribute to blastocysts with a greater proportion of epiblast cells than alpha blastomeres do and that vegetal blastomeres, which exhibit reduced developmental potential, are more frequently classified as alpha. This study provides the first evidence of intrazygotic and interblastomere proteomic asymmetry in mammals, indicating its role in early lineage segregation. We anticipate that a high-resolution, multimodal integrative platform capable of simultaneously capturing cellular identity, localization, and functional state can advance our understanding of developmental biology to a high degree.

### Biomarker discovery and validation

Recently, multiple reviews have consistently highlighted that multimodal biomarkers—from blood or tissue to spatial proteomics—are driving advances in early disease detection, prognostic stratification, and precision therapeutic decision-making [182–185]. Traditional high-throughput omics platforms lack spatial resolution, hindering the precise localization of candidate biomarkers and obscuring their tissue-specific mechanisms [186, 187]. Spatial methods such as IHC provide spatial information at the cellular level, improving diagnostic precision (Table 5). Multiplexed imaging platforms, including IMC and MIBI, can simultaneously detect more than 40 markers in a single tissue section, yielding a detailed view of cell–cell interactions and intratumoral heterogeneity. Compared with traditional qualitative assays, these quantitative approaches generate richer data for identifying biomarkers that predict disease recurrence [183].

**Table 5:** Applications of MS-based spatial omics for biomarker discovery and validation

MS method	Disease type	Targets	Main findings	References
MALDI-MSI	Neuroblastoma	Glycome	MYCN expression upregulates GMDS, promoting core fucosylation and driving tumor progression and growth	Zhu et al. (2025) [169]
MALDI-MSI	LUSC	Metabolome	ABP1, PLA2G1B, and SNAI1 are associated with poorer prognosis	Wang et al. (2025) [170]
MALDI-MSI	Glioblastoma	Proteome, lipidome	Lipid ions at m/z 864.7, 866.7, and 881.7 were identified as markers of long-term survival (> 36 months), whereas ions at m/z 760.6, 788.6, and 810.6 were associated with shorter survival durations (< 30 months). Among prognostic protein markers, ANXA6 and GPHN were linked to poor outcomes, while RPS14 and MTDH were associated with favorable prognosis	Lagache et al. (2025) [171]
MALDI-MSI	Lung cancer	Metabolome	GUK1 phosphorylation shows strong spatial correlation with guanine nucleotide enrichment in ALK⁺ lung cancer tissue	Schneider et al. (2025) [205]
MALDI-MSI	Melanoma	Proteome	SIRT1 has potential as both a prognostic and predictive biomarker for response to α-PD-1 immunotherapy	Placke et al. (2025) [206]
MIBI-TOF	Alzheimer’s disease	Proteome	Neuronal MFN2 expression near tau pathology may confer a survival advantage, highlighting its potential as a biomarker in AD	Vijayaragavan et al. (2022) [209]
DVP	Toxic epidermal necrolysis	Proteome	Activation of type I/II interferon signatures and phosphorylated STAT1 represents a key pathogenic signal in TEN	Nordmann et al. (2024) [210]
MALDI-MSI	Diabetic kidney disease (DKD)	Metabolome	Kidney Precision Medicine Project (KPMP) spatial metabolomics identified adenine as a spatially localized biomarker linked to tubular injury and kidney dysfunction	Sharma et al. (2023) [121]

For example, as previously mentioned, high MYCN expression upregulates GDP-mannose 4,6-dehydratase (GMDS), enhancing core fucosylation to cause tumor progression and growth [169]. The MALDI-MSI technique revealed the mechanism underlying MYCN amplification associated with core fucosylation, helping us to identify GMDS as a potential target treatment strategy for MYCN amplification-type neuroblastoma. Additionally, by integrating metabolite–gene–immune information, Wang et al. identified potential biomarkers at three levels: the metabolic, transcriptomic, and subtype levels [170]. At the metabolic level, they reported that the use of phosphatidylcholines as metabolites has an unfavorable effect on the progression-free survival (PFS) of lung squamous cell carcinoma (LUSC) patients. They also reported that ABP1, PLA2G1B and SNAI1 were linked to poor prognosis, highlighting their potential as immune-related molecules at the transcriptomic level. Moreover, on the basis of mass spectrometry-defined metabolic tumor subpopulations (MTSs), patients can be classified into groups with distinct treatment strategies. Thus, clustering patients by mass-based metabolic heterogeneity provides a promising biomarker for stratification for personalized therapy. In addition, through a dry proteomics workflow, Lagache et al. reported several potential lipid ions and protein biomarkers that are associated with patient prognosis in patients with glioblastoma [171]. They reported lipid ions with m/z values of 864.7, 866.7, and 881.7 as markers for long-term survival (> 36 months), whereas lipid ions such as m/z 760.6, 788.6, and 810.6 were associated with shorter survival durations (< 30 months). Moreover, this workflow revealed prognostic protein markers, with ANXA6 and GPHN linked to unfavorable outcomes and RPS14 and MTDH associated with favorable prognosis. Notably, the lipid and protein biomarkers identified in this study are the same as those in previous studies [188, 189].

Beyond these cancer-focused biomarker studies, spatial metabolomics has also started to show clear clinical relevance in large human research consortia. One representative example is the NIH Common Fund-supported Kidney Precision Medicine Project (KPMP), which applies MSI-based spatial metabolomics to clinically annotated human kidney biopsy samples to study chronic kidney disease (CKD) and diabetic kidney disease (DKD). In KPMP studies, adenine and related purine metabolites were identified as spatially localized molecular features associated with tubular injury and impaired kidney function [121]. These metabolites therefore serve not only as mechanistic indicators but also as candidate biomarkers for disease stratification. Importantly, these results were derived from patient tissues and validated across multiple cohorts, highlighting the translational potential of MSI for clinically oriented biomarker discovery.

For biomarker discovery, future work will include the standardization of analytic workflows and biomarker quantification. Integrating artificial intelligence (AI)-driven analysis with multimodal data, such as transcriptomics, digital pathology, and clinical information, will enable the discovery of more precise biomarkers.

### Precision medicine approaches

Precision medicine delivers individualized treatment by accounting for the heterogeneity of the same disease [190]. With the utilization of individual genomic, lifestyle, and environmental information, precision medicine enables the development of biomarkers for early disease detection, monitoring disease progression and providing targeted therapeutic strategies [191]. Although traditional bulk or single-cell analysis can be used to detect the dynamics of proteins, understanding the location of changes in tissue is difficult, as it is difficult to determine cell‒cell interactions and disease mechanisms and develop therapeutic strategies [62, 192]. Spatial omics methods integrate proteomics, lipidomics, and metabolomics with pathology, enabling direct visualization of molecular alterations at pathological sites. These approaches reveal molecular mechanisms within cells and their microenvironment, providing insights into disease progression and immune responses. Through high-throughput spatial imaging technologies and MS-based methods, multiple protein segmentations and metabolites can be detected in a single tissue section, facilitating the identification of potential predictive biomarkers from specific pathological regions or cell types (Table 6).

**Table 6:** Applications of MS-based spatial omics for precision medicine

MS method	Disease type	Targets	Main findings	References
MALDI-MSI	LUSC	Metabolome	Seven metabolic tumor subpopulations displayed distinct immune infiltration patterns and varying sensitivity to chemotherapy	Wang et al. (2025) [170]
MALDI-MSI	Glioblastoma	Proteome, lipidome	The ‘dry proteomics’ analysis workflow integrates machine learning with multi-omics data to characterize tumor heterogeneity, identify biomarkers, classify functions, and predict prognosis	Lagache et al. (2025) [171]
MALDI-MSI/DESI-MSI	Renal cell carcinoma	Metabolome, lipidome	Co-treatment with AZD2014 and AZD8186 (a PI3Kβ inhibitor) reduced the non-responder regions observed in AZD2014-treated PTEN-null renal cell carcinoma.carcinoma tumor tissue	Ling et al. (2025) [193]
DVP	Toxic epidermal necrolysis	Proteome	The JAK/STAT and interferon pathways represent promising therapeutic targets in TEN, with JAK inhibitors showing potential efficacy	Nordmann et al. (2024) [210]

For example, by a combination of MS-based spatial metabolomics and IHC, LUSC patients were clustered into seven metabolic tumor subpopulations (MTSs). Each MTS exhibited distinct profiles of immune infiltration and chemotherapy sensitivity, providing different precision treatment strategies for each subpopulation [170]. Additionally, the dry proteomics workflow, which integrates machine learning with multiomics data, enables tumor heterogeneity characterization, biomarker identification, functional classification and prognosis prediction [171]. In renal cell carcinoma, combining AZD2014 with AZD8186 (a PI3Kβ inhibitor) reduced the non-responder regions of AZD2014 (an mTORC1/2 inhibitor)-treated PTEN-null renal cell carcinoma tumor tissue, indicating enhanced pathway inhibition. These findings provide a new strategy for precision treatment of renal cell carcinoma [193]. MS-based spatial proteomics holds great promise for precision medicine, yet future efforts should focus on enhancing spatial resolution, standardizing workflows, and integrating multimodal data to enable clinically actionable insights.

In summary, MS-based spatial omics provides powerful insights into the spatial organization of proteins, metabolites, lipids, and other molecules within tissues, enabling advances in human biology and precision medicine. Beyond the examples discussed above, MS-based spatial methods can also be applied to drug discovery, infectious disease research, and immunology. In the future, the integration of multispatial omics data and clinical validation will be crucial.

## Challenges and future directions

Over the past decade, the convergence of mass spectrometry and spatial omics has accelerated dramatically, driven by instrument advancement, cross-modal AI implementation, and cloud computing. However, three gaps, (1) spatial resolution and sensitivity, (2) data standardization and explainability, and (3) clinical validation, still separate laboratory discoveries from real-world adoption.

### Spatial resolution and sensitivity

The laser spot in conventional MALDI often exceeds the organelle dimensions, restricting MSI to pixels of a few micrometers. The combination of MALDI-2 with the oversampling method, where each pixel partially overlaps its neighbors, improves the resolution to below 2 µm and the sensitivity for various metabolite classes (e.g., amino acids, purines, and carbohydrates), although at the cost of longer acquisition times and larger file sizes [66, 67]. SIMS narrows the spot further to 50–150 nm, but low ion counts, restricted chemical coverage, and surface damage remain challenging. Additionally, ion suppression and matrix effects frequently bury signals of low-abundance molecules or posttranslational species. Ion mobility spectrometry (IMS), such as trapped ion mobility spectrometry (TIMS) and high-field asymmetric waveform ion mobility spectrometry (FAIMS), increase the peak capacity and signal-to-noise ratio, improving the detection of lipids, metabolites, and even proteoforms [76, 91, 194, 195]. Looking forward, the introduction of plasmonic or catalytic nanomaterials, including gold nanoparticles (AuNPs) and fluorinated AuNPs, titanium dioxide nanowires, and 2D nanoflake-capped silicon nanowires (SiNWs), is anticipated to increase the resolution and sensitivity of MSI [196–198].

### Data standardization and explainability

Diverse MS platforms generate petabytes of heterogeneous spectra, but community-wide standards for quality control and batch correction are still under development. A recently proposed quality control standard (QCS) offers reference materials and metrics that reduce interbatch variance and provide a shared benchmark [199]. Moreover, data discoverability and reuse are limited because (1) public MSI repositories remain sparse, (2) metadata schemas are inconsistent, and (3) clinical datasets face the difficulties of consent and deidentification, making it laborious to aggregate, search, and validate results across studies. Interlaboratory variation in sample preparation, instrumentation, and data processing further increases variance, highlighting the importance of data reproducibility and multicenter harmonization.

As introduced above in the context of MSI segmentation, the black-box nature of deep learning MSI methods constrains clinical trust, even if such models can already segment tissues and identify representative m/z bins. Explainable artificial intelligence (XAI) has therefore emerged to provide transparent and interpretable explanations for AI-driven MSI outputs [200]. While a deep learning model may accurately identify a tumor region, XAI tools can reveal which specific molecular features or m/z peaks the model used to make that determination. Popular methods for spectroscopic models include SHapley Additive exPlanations (SHAP), local interpretable model-agnostic explanations (LIME), and class activation mapping (CAM) [138, 201, 202]. These XAI tools help researchers verify whether a model focuses on chemically meaningful features, uncovers hidden diagnostic peaks, and increases confidence when AI pipelines are implemented in research or clinical settings. By bridging the gap between complex AI decisions and human understanding, XAI is essential for the clinical translation of AI-driven MSI workflows.

### Clinical translation and cost barriers

MSI methods have evolved from laboratory technologies to proof-of-concept systems with clinical potential. SpiderMass, an instrument allowing real-time MS analysis under in vivo conditions with minimal damage, was converted into a robot-guided, 3-D topographic MSI platform, opening a new avenue for real-time, minimally invasive molecular imaging on living or intact biological surfaces for surgical application to excision margins [203, 204]. However, wide clinical application of MSI remains challenging, mainly because of costs and human resources. High-resolution instruments cost several hundred thousand or even more than 1 million USD, while reagent and consumable expenses increase sharply in high-throughput studies. Moreover, several months may be needed to train a specialist to operate such systems. Consensus standard operating procedures for sample handling, matrix deposition, and data processing are therefore essential before MSI can become routine.

Together, the coordinated advancements in hardware, software, and workflow economics will transform MS-based spatial omics from a specialized laboratory technique into a broadly implemented tool for clinical applications, especially biomarker discovery and precision diagnostics.

## Conclusion

In this review, we depict a comprehensive landscape of MS-based spatial omics that ranges from technical and algorithmic advances to integrative frameworks and applications, and we trace its trajectory from early technical foundations to current multimodal and high-throughput platforms with emerging clinical potential. This new molecular cartography redefines our understanding of tumor microenvironments, neurodegeneration, and developmental biology while building a direct bridge from basic science to clinical applications such as biomarker discovery and precision therapy. Innovation across hardware, software, and translational medicine, together with the creation of a standardized molecular atlas, should propel MS-based spatial omics into routine clinical practice within the next decade. We therefore call on the community to share code and raw data, build universal standards, establish clear validation pipelines, and train interdisciplinary researchers with expertise in biology, MS, and AI. Spatial omics has moved MS beyond the question of “what is present” to reveal “where it is and how it interacts”; continued advancements will turn MS into a mainstream approach for purposes ranging from understanding biological complexity to realizing personalized medicine.

## Data Availability

No datasets were generated or analysed during the current study.

## Abbreviations

3D-SSNet: 3D sparse sampling network
Aβ: Amyloid-beta
AD: Alzheimer’s disease
AFADESI‒MSI: Air-flow-assisted desorption electrospray ionization-mass spectrometry imaging
AI: Artificial intelligence
AP-MALDI: Atmospheric-pressure MALDI
CAM: Class activation mapping
CCS: Common coordinate system
cGAN: Conditional generative adversarial network
CNS: Central nervous system
CT: Computed tomography
DAN: 1,5-Diaminonaphthalene
DANDI: Distributed Archives for Neurophysiology Data Integration
DESI: Desorption electrospray ionization
DHAP: Dihydroxyacetophenone
DLADS: Deep learning approach for dynamic sampling
DOMs: Dynamic organelle maps
DUV: Deep ultraviolet
DVP: Deep visual proteomics
ECM: Extracellular matrix
ERD: Expected reduction in the distortion
ESI: Electrospray ionization
EUV: Extreme ultraviolet
FAIMS: High-field asymmetric waveform ion mobility spectrometry
FFPE: Formalin-fixed paraffin-embedded
FTICR: Fourier-transform ion cyclotron resonance
FTIR: Fourier transform infrared microscopy
GAG: Glycosaminoglycan
GAMSI: Gel‑assisted mass spectrometry imaging
GMDS: GDP-mannose 4,6-dehydratase
H&E: Hematoxylin and eosin
HATU: Hexafluorophosphate azabenzotriazole tetramethyl uronium
hiPSC: Human induced pluripotent stem cell
HuBMAP: Human Biomolecular Atlas Program
IHC: Immunohistochemistry
IMC: Imaging mass cytometry
IMS: Ion mobility spectrometry
ISH: In situ Hybridization
ITO: Indium tin oxide
KPMP: Kidney Precision Medicine Project
L1CAM: L1 cell adhesion molecule
LCM–MS: Laser capture microdissection coupled mass spectrometry
LC‒MS/MS: Liquid chromatography‒tandem mass spectrometry
LDI: Laser desorption/ionization
LIME: Local interpretable model-agnostic explanations
LOPIT: Localization of organelle proteins by isotope tagging
LUSC: Lung squamous cell carcinoma
MALDI: Matrix-assisted laser desorption/ionization
MALDI-IHC: MALDI-immunohistochemistry
MALDI-ISH: MALDI-based in situ hybridization
MI: Mutual information
MIBI: Multiplexed ion beam imaging
microPOTS: Microdroplet processing in one pot for trace samples
MIMIC: Mass imaging modality integration coregistration
MIPI: Metabolome-informed proteome imaging
MOSR: MSI from optical super-resolution
MRI: Magnetic resonance imaging
MS: Mass spectrometry
MSC: Microglial state continuum
MSI: Mass spectrometry imaging
MTS: Metabolic tumor subpopulation
N2V: Noise2Void
nanoPOTS: Nanodroplet processing in one pot for trace samples
NCC: Normalized cross-correlation
ND: Neurodegenerative disease
NGS: Next-generation sequencing
NPC: Neural progenitor cell
OTCD: On-tissue chemical derivatization
PC: Principal component
PCA: Principal component analysis
PEG: Polyethylene glycol
PFS: Progression-free survival
PIMS: Python image management system
QCL-MIR: Quantum cascade laser mid-infrared microscopy
QCS: Quality control standard
ROC: Receiver operating characteristic
ROI: Region of interest
SHAP: Shapley additive explanations
SIMS: Secondary ion mass spectrometry
SiNW: Silicon nanowire
STB: Syncytiotrophoblast
TCPP: Tetrakis(4-carboxyphenyl)porphyrin
TEMI: Tissue‑expansion mass spectrometry imaging
TEM-NanoSIMS: Transmission electron microscopy and nanoscale secondary-ion mass spectrometry
TIC: Total ion current
TIMS: Trapped ion mobility spectrometry
TIL: Tumor-infiltrating T lymphocyte
TOF: Time-of-flight
TME: Tumor microenvironment
TMPA: Tris(2-pyridylmethyl)amine
t-SNE: T-distributed stochastic neighbor embedding
VAE: Variational autoencoder
XAI: Explainable artificial intelligence
